# 1797. Pneumococcal Vaccination in Veterans 65 years and older with Pneumococcus in the Veterans Health Administration (VHA) since November 2021

**DOI:** 10.1093/ofid/ofad500.1626

**Published:** 2023-11-27

**Authors:** Patricia Schirmer, Cynthia A Lucero-Obusan, Gina Oda, Mark Holodniy

**Affiliations:** Department of Veterans, Greenwood Village, Colorado; U.S. Department of Veteran Affairs, Public Health National Program Office, Palo Alto, California; Department of Veterans Affairs, Palo Alto, CA; Department of Veterans Affairs, Palo Alto, CA

## Abstract

**Background:**

Pneumococcal infection is a vaccine-preventable illness caused by *Streptococcus pneumoniae*. In October 2021, the Advisory Committee on Immunization Practices updated pneumococcal vaccine guidelines in all adults >65 years and for adults 19-64 years with certain medical conditions or risk factors. Pneumococcal vaccination among patients >65 years is a national VHA performance measure. We reviewed vaccine status in patients >65 years with pneumococcal infection since guidelines changed.

**Methods:**

Demographics, pneumococcal vaccination in relation to time of infection and laboratory testing (culture, molecular and urinary antigen) during 11/1/2021-4/11/2023 were retrieved from VHA data sources. Patients aged >65 years with pneumococcus were evaluated and compared to VHA’s electronic quality measurement (eQM) for national pneumococcal vaccination rates.

**Results:**

1,128 cases (1,108 unique patients) were identified (Table 1). 20 patients had recurrent infections. Median age was 74 years (IQR 69-77) with non-Hispanic Blacks (14.1%) slightly overrepresented compared to VHA’s Black population (12.7%); primary infection sites were pulmonary (68.4%) and blood (23.5%). 1,006/1,128 (89.2%) of cases in patients >65 years had at least 1 pneumococcal vaccine. VHA’s national pneumococcal vaccination of patients >65 years for fiscal years 2022-23 was 65.3%-65.9% per eQM. Of cases not vaccinated, 22.1% were non-Hispanic Black patients. Median time of vaccination before infection was 4.7 years (IQR 2.8-6.5). Most recent vaccination prior to infection for the overall cohort and those with recurrent infections was 23-valent pneumococcal polysaccharide vaccine (PPSV23) (56.5% and 57.9% respectively). In addition, 108/1,128 (9.6%) cases were vaccinated after their pneumococcal infection (17/108 were not previously vaccinated). 71/108 (65.7%) vaccines given after illness were 20-valent pneumococcal conjugate vaccine (PCV) (PCV20).

**Figure:**
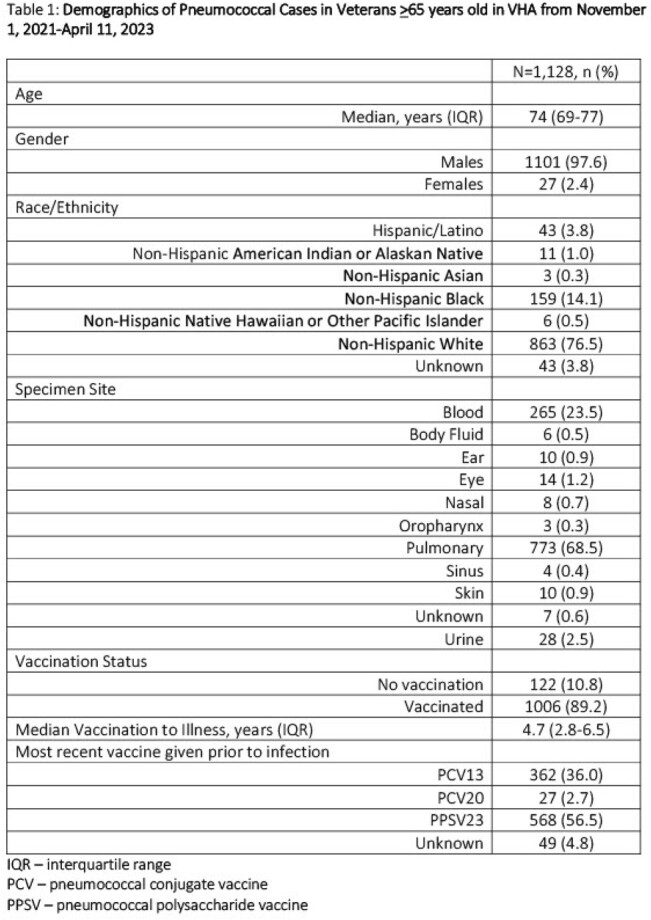

**Conclusion:**

In VHA patients >65 years with pneumococcal infection, nearly 90% had had at least 1 pneumococcal vaccine which was mainly PPSV23 and given on average almost 5 years before infection. VHA could target non-Hispanic Black patients with pneumococcal vaccination efforts and ensure providers are utilizing PCV20 or PCV15+PPSV23 vaccines.

**Disclosures:**

**All Authors**: No reported disclosures

